# Placenta and Placental Derivatives in Regenerative Therapies: Experimental Studies, History, and Prospects

**DOI:** 10.1155/2018/4837930

**Published:** 2018-01-18

**Authors:** Olena Pogozhykh, Volodymyr Prokopyuk, Constança Figueiredo, Denys Pogozhykh

**Affiliations:** ^1^Institute for Transfusion Medicine, Hannover Medical School, Carl-Neuberg-Straße 1, 30625 Hannover, Germany; ^2^Institute for Problems of Cryobiology and Cryomedicine, National Academy of Sciences of Ukraine, Pereyaslavskaya Str. 23, Kharkov 61015, Ukraine

## Abstract

Placental structures, capable to persist in a genetically foreign organism, are a natural model of allogeneic engraftment carrying a number of distinctive properties. In this review, the main features of the placenta and its derivatives such as structure, cellular composition, immunological and endocrine aspects, and the ability to invasion and deportation are discussed. These features are considered from a perspective that determines the placental material as a unique source for regenerative cell therapies and a lesson for immunological tolerance. A historical overview of clinical applications of placental extracts, cells, and tissue components is described. Empirically accumulated data are summarized and compared with modern research. Furthermore, we define scopes and outlooks of application of placental cells and tissues in the rapidly progressing field of regenerative medicine.

## 1. Background

The human placenta is a unique temporary organ which ensures mutual coexistence of the organisms of mother and fetus, determining growth and development of the latter [1]. Initially, it was believed that the fetus and placenta are closely related genetically to the mother; but with the development of assisted reproductive technology of the egg donation, it became clear that their genotypes could be completely foreign [2], which can be regarded as a natural model of engraftment after allogeneic transplantation. The main functions of the placenta are ensuring the supply, growth, and development of the fetus, as well as removing metabolic products and preventing immune rejection [1]. Since the placenta is a provisional organ, it becomes a salvage material after delivery [3]. For decades, clinicians and researchers work on the application of the placenta for therapeutic purposes, initially in the form of extracts and cell or tissue transplants, thus accumulating substantial empirical experience [4, 5]. However, at the same time, a large amount of research was little systemized and not always correlated with conventional pharmaceutical and other methods of treatment. Recent developments of cell therapy approaches along with opportunities for autobanking significantly increased the interest in the placenta as a source of biological material. Novel studies revealed a number of typical features of placental-derived cells, which define the direction of clinical use [6]. The major aim of this review was to identify and systemize general properties specific to various biological products of placental origin and characterize the most promising directions for their clinical application based on the analysis of data available in the scientific literature. Since placental structures have been used in a broad range of therapies, in our analysis, we have only considered the data, which have been confirmed repeatedly by several independent groups at various time points.

## 2. Structure and Properties of the Placenta and Fetal Membranes

### 2.1. Development of the Placenta

**Figure 1:**
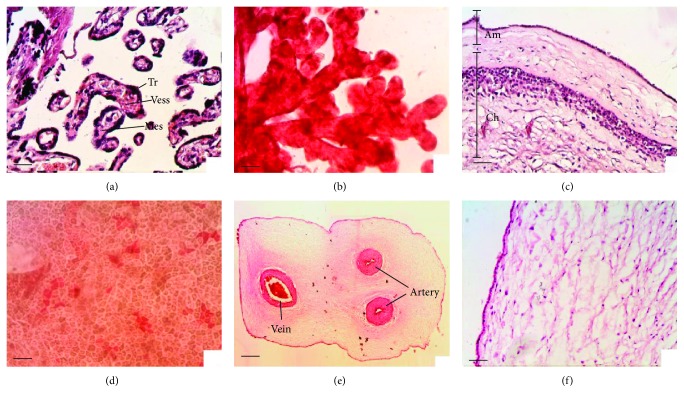
Morphology of placental components: (a, b) placental tissue (villi): Tr: trophoblast; Vess: vessels; Mes: mesenchyme; (с) fetal membranes: Am: amniotic membrane; Ch: chorionic membrane; (d) surface cells of amniotic epithelium; (e) cross-section of the umbilical cord; (f) umbilical cord tissue. Staining: (a, c, e, and f) hematoxylin-eosin, sections; (b, d) neutral red, native preparation. Scale bars: (a, b, c, d, and f) 50 *μ*m; (e) 1000 *μ*m.

During pregnancy provides the key to understand its structural and functional peculiarities. At the stage of 8 blastomeres, the blastocyst divides into embryoblast and trophoblast. Trophoblast forms villi and first primary, containing only the trophoblast, then secondary, containing the stroma (embryonic mesenchyme), and later tertiary, containing the vessels (Figures 1(a) and 1(b)). At the same time, division of the trophoblast into syncytium and cytotrophoblast takes place. Implantation processes and trophoblast invasion occur through the enzymatic destruction of the endometrium and decidua of the uterus and layering of the resulting lacunae with trophoblast cells, replacing the choroid of the spiral arteries with trophoblast, which prevents thrombosis and makes the arteries refractory to vasopressor agents. After 6–8 weeks of pregnancy, the villi remain only on the placental site. The rest of the villi become atrophied and the smooth chorion, containing significant amounts of the trophoblast elements, is being formed [6, 7].

### 2.2. Morphology

Postpartum placenta has a disk-shaped form 16–20 cm in diameter, weighing 500 g on average. Trophoblast cells, mesenchymal cells, and endothelial cells of vessels are the main cell types of the placenta.

Since the use of “early placenta” (from the first two trimesters of pregnancy) encounters a number of ethical issues, the majority of the researchers are focused on the third-trimester placenta, also known as “mature placenta” (38–40 weeks of pregnancy) [3, 8]. Mature placenta consists of fetal and maternal parts. The fetal part includes the chorionic plate, amnion, and umbilical cord. Fetal membranes (Figure 1(c)), amniotic and chorionic, are formed on the basis of the smooth chorion and can be easily separated at the intermediate layer. Thin, transparent, and smooth amniotic membrane is composed of a single layered epithelium and the amniotic mesenchyme, an avascular connective tissue. Chorionic membrane is composed of fibroblasts and a large number of trophoblast cells. The chorionic plate represents the fetal surface of the placenta, which is covered by the amnion. The umbilical cord enters the chorionic plate and connects the fetus to placenta. Umbilical cord is 50–70 cm in length and 1-2 cm in thickness. It is covered by the amniotic epithelium and contains two arteries and one vein that are immersed in the Wharton's jelly (which contains a large amount of fibroblast cells and has an intercellular substance rich in hyaluronic acid) (Figures 1(d)–1(f)) [1, 6]. The maternal part, or so-called basal plate, is comprised of bed and walls of lacunae, formed by decidual endometrial tissue. Additionally, the maternal part contains NK cells, macrophages, and other immune cells. Therefore, postpartum placental cells possess mainly the fetal genotype with a certain amount of maternal cells [1, 7].

### 2.3. Immunological Features

The structure of the placenta has several features that determine its function as well as the possibility of effective application in clinics and in biotechnology. Trophoblast cells are protected from the maternal immune system, due to reduced expression of the major histocompatibility complex (MHC), apoptosis-inducing mechanisms, and the influence of hormones and growth factors on the cells of the immune system [9]. According to majority of the authors as well as to online gene annotation portals and databases (e.g., http://biogps.org/), trophoblast has virtually no expression and other cells of placenta express very low amounts of classical MHC (HLA-A, HLA-B, and HLA-C), thus making it difficult for the immune system to recognize these cells [10, 11]. Furthermore, trophoblast expresses nonclassical MHC, which is inherent to pregnancy and includes HLA-E, HLA-F, and HLA-G [2]. In particular, according to some authors, HLA-G inhibits the migration of natural killer cells (NK cells) and proliferation of T-lymphocytes by interacting with the NKR2B4 receptor. Similar properties were described for the HLA-E expressed in the trophoblast [2, 7, 12].

Other mechanisms of protection of trophoblast from the maternal immune aggression involve apoptosis-inducing ligands FasL and TRAIL, which have the influence on immunocompetent cells [13]. During pregnancy, an increase in the number of Th2 lymphocytes occurs, which secrete anti-inflammatory interleukins IL4, IL5, IL9, IL10, and IL13, and a decrease in Th1 lymphocytes, which secrete proinflammatory IFN*γ*. This phenomenon is determined by the action of progesterone, as well as the capability of placental cells to secrete a certain variety of cytokines. The reduction in NK cells is also detected, though it is compensated by the activation of nonspecific immunity [2]. In this regard, it is observed that pregnancy is often accompanied by remission of a number of autoimmune diseases, such as multiple sclerosis and rheumatoid arthritis [2, 14].

### 2.4. Endocrine Function

Trophoblast cells synthesize a number of hormones, such as estradiol, progesterone, and chorionic gonadotropin, which regulate growth and development of the fetus as well as changes in the organism of a mother during pregnancy [15]. These hormones have the impact primarily on the reproductive and immune systems, which explains the therapeutic efficiency of the components of the placenta in the treatment of respective pathologies. Estradiol causes proliferation of the endometrium and mammary glands, causes calcium retention, and possesses feminizing and antisclerotic effect, as well as it affects sexual behavior. The main function of progesterone is in providing occurrence and preservation of pregnancy. Chorionic gonadotropin is an analogue of gonadotropin hormones, with the properties of luteinizing and follicle-stimulating hormones. It possesses the trophic corticotropic function and supports the development of pregnancy as well as enhanced resistance to stress. Exogenous administration of human chorionic gonadotropin stimulates ovulation, synthesis of ovarian estrogen in females, and androgen synthesis and spermatogenesis in males [15, 16].

### 2.5. Trophoblast Deportation

Among the most important physiological properties of placental cells is the capability of deportation and long-term existence in the mother's organism outside of the placenta itself. Fragments of different sizes, from multinuclear symplasts to exosomes, are emitted from the villi and fall into the uterine veins; a part of them are embolized by pulmonary capillaries and smaller particles fall in the systemic circulation. Settling in the mother's tissues, trophoblast cells can remain viable for some time and can be traced up to three–four days on average, with certain cases of reported detection in up to two weeks postpartum [17]. Elimination of deported trophoblast cells is achieved through nonspecific immunity and lytic factors. To date, there is no consensus about the role of trophoblast deportation in the course of pregnancy. Most researchers believe that this is a physiological phenomenon and assumes that the trophoblast in such manner participates in the formation of tolerance [17, 18]. In the case of preeclampsia and placental dysfunction, the amount of necrotic and apoptotic-altered trophoblast cells increases. In modern medicine, diagnostic methods based on the isolation of fetal cells from the maternal blood are actively developing. At the moment, this is primarily a diagnosis of hereditary diseases and risk of preeclampsia. Therefore, not only the placenta is a natural model of organ transplantation in the organism of a mother during pregnancy but also the trophoblast deportation is a natural model of cell transplantation [12].

## 3. History of Clinical Application of Placental Components

Multicomponent cellular and biochemical composition of the placenta, along with its ability to perform a wide variety of functions and availability of a large amount of material, has always attracted close attention of clinicians and researchers. Substantial experience in the application of the placenta in experimental and clinical practice has accumulated over the past 100 years. Various groups of researchers at different time points often observed similar therapeutic effects and patterns even by applying different techniques and using different dosages and forms (extracts, tissue fragments, cells, serum, etc.) [4, 8, 16, 19]. Unfortunately, this immense amount of work and clinical data is little systemized and many successful approaches received no follow-up. We believe that analysis of these data will contribute to finding ways of further development of therapies using the placental material.

The first application of the amniotic membrane in ophthalmology was published by Davis, J. (1910) [8]. Results of the first studies on the effect of placental extracts and tissues on the reproductive system appear in the literature in the early 20th century (Aschner B., 1912, Hirose T., 1919) [16]. Majority of the world's research on devitalized preparations was published in the 1930s to 1980s. The first successful transplantation of cord blood cells in Fanconi anemia in 1982 resulted in enhanced interest of researchers in cord blood stem cells [20]. Since the end of the 20th century, components of the placenta (placenta, umbilical cord, and membranes) are increasingly seen as a source of stem cells; cryopreserved viable placental preparations are successfully applied [6, 21].

Due to a range of historical reasons, a significant amount of the studies is unfortunately not included into modern international databases, such as PubMed. For example, most of the works of academician Vladimir P. Filatov [22], founder of the Institute of Tissue Therapy and Eye Diseases (USSR), in the 1930s to 1960s were not even translated to English, highly limiting the audience of this valuable publications. At the same time, his fundamental work on the use of tissue therapy methods has been published in over 3000 scientific works; thousands of patients received effective treatment with devitalized placental medications; and dozens of departments and institutes on tissue therapy were established on this basis [23–25]. Also, currently, more information on the centuries of experience of application of placental derivatives in traditional Chinese medicine becomes available from the recent publications [8].

**Figure 2:**
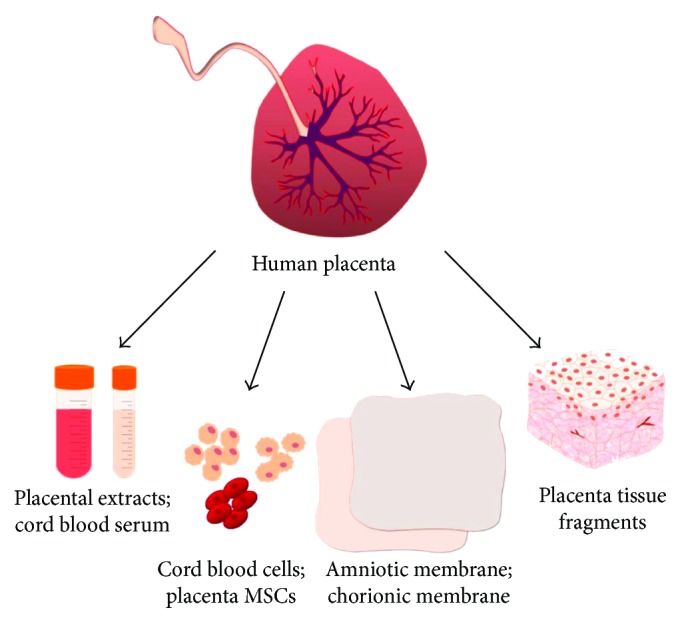
Conventional forms of application of placenta-derived biomaterial in clinics: placental extracts and lyophilizates, cord blood serum, various types of differentiated and stem cells, amniotic and chorionic membranes, and fragments of placental tissue.

The researchers have used in their studies the fragments of placental tissue, amniotic and chorionic membranes, umbilical cord, amniotic fluid, placental extracts and lyophilizates, and cord blood serum, as well as various types of differentiated cells and stem cells (Figure 2). Such material has been used in native form as well as after chemical and thermal processing, after cryopreservation and sublimation. Methods of application widely vary such as subcutaneous, intramuscular, intravenous, intraorperative, as biocovers, and substitutive material, as well as via oral administration [26–32].

The views on the mechanism of action of placental preparations have been changing along with the development of biology and medicine. The researchers hypothesized and attempted to explain the patterns of therapeutic effects by vitamin, mineral, protein, and peptide composition, by the presence of “resistance factors,” “preservation factors,” cytokines, hormones, and stem cells [4, 21, 23]. Such approach could explain common features of placental derivatives and shed light on the mechanism aspect and unique biological activity. At the same time, very often, the studies of the beginning of 21st century repeat the works from the mid-twentieth century, explaining obtained results in a different manner, while observing similar effects under the same pathological conditions [8, 19].

## 4. Cord Blood Cells

The greatest attention of researchers and clinicians is traditionally attracted by cord blood cells. Umbilical cord blood is an easily accessible rich source of hematopoietic stem cells which is an alternative to the bone marrow [33–35]. High rates of success were achieved with allogenic transplantation of umbilical cord blood cells for the treatment of the patients with hematologic and metabolic pathologies [20, 36]. Application of cord blood stem cells is described to be quite effective in the treatment of erectile dysfunction and diabetes mellitus, resulting in improved erectile function and reduction in fasting blood sugar [37]. In former years, mainly due to the limited dosage of cord blood cells available for each transfusion, the application was limited to child patients [38]. However, recent advances in cord blood cell expansion methods, isolation of particular units with the following dosage adaptation, and availability of HLA matching techniques allowing pooling of the units from the different donors resulted in significant improvement of related therapies [20, 39–42].

Besides the transplantation of hematopoietic cord blood cells, a special interest of the researchers is attracted by erythrocytes and platelets of the cord blood [43–45]. The main distinction of cord blood erythrocytes from the erythrocytes of an adult is the content of fetal hemoglobin. The content of fetal hemoglobin in cord blood erythrocytes is over 90%, while in adult blood it is less than 10% [46]. Fetal hemoglobin has a significantly higher affinity to oxygen, which enables the transfer of oxygen through the placenta from the mother's blood [47]. Despite the possibility to obtain only 50–100 ml of cord blood postpartum, transfusion of the cord blood erythrocytes is advantageous for the treatment of infants, especially premature infants, as well as for the intrauterine blood transfusions, when the recipient's blood mainly contains the fetal hemoglobin. Such approach allows reducing the number of transfusions and significantly accelerates recovery [48, 49]. Transfusion of the cord blood erythrocytes to adult patients results in a more rapid recovery of neutrophils and slower recovery of platelets in comparison to transfusion of adult erythrocytes [42]. Transfusion of the cord blood is also mentioned as a perspective for utilization in pediatric practice in the countries where the availability of donated blood is limited [33].

## 5. Placental Extracts

High number of publications is related to the studies of placental extracts. Such extracts are obtained by lysing human placental tissues collected at full-term delivery. Therefore, the extracts do not contain cells but are rich in a wide range of proteins, minerals, amino acids, and steroid hormones [4]. According to the data of various research groups, such extracts possess anti-inflammatory, analgesic [26], antioxidant [50, 51], cyto- and radioprotective [52], and anti-allergic properties and express hormonal activity [16, 53, 54], as well as stimulate proliferation and reparative processes [14, 55, 56].

Placental extracts were shown to enhance the proliferation of fibroblasts and cord blood cells *in vitro*. At the same time, it was noted that extracts isolated from the late gestation placenta possess the highest biological activity [53, 56]. Cytoprotective and antioxidant properties of the extracts are usually associated with the protein components; in particular, they are correlated with the concentration of alpha-fetoprotein [50, 51]. Animal model studies showed that prophylactic administration of the extracts increases the resistance of animals to oxidative stress [57]. Placental extracts reduce the concentration of free radicals, inflammatory cytokines IL6, TNF, and IL1, at the same time increasing the colony formation of progenitor cells *in vitro* and reducing oxidative and radiation damage of the cells [52, 57]. Analysis of biosafety of placental extracts revealed the absence of toxic or mutagenic influence on cell cultures and adult animal models; however, fetotoxicity in animals at early gestation was reported [58].

Placental extracts have been applied for the treatment of a wide variety of pathological conditions—most commonly in surgery, neurology, gynecology, and dermatology. Pronounced positive effects were received in the treatment of wounds, nonhealing ulcers, and burns: rate of epithelialization was significantly increased and a decrease of infiltration and reduction of the pain syndrome were observed [27, 59]. The extracts accelerate the wound healing in animals with the diabetes model, which can be interpreted as a treatment for diabetic neuropathy and angiopathy [28]. The mechanism of action of placental extracts in the wound healing is associated with the increase of TGF-*β* in the early phase of regeneration and VEGF in the late phase, as well as with the presence of FGF, amplification of angiogenesis, and the increase of expression of CD31 [28, 60]. Application of placental extracts in menopausal disorders allowed reducing the number and severity of hot flushes, irritability, and normalize hormonal profile [54, 61]; the amount of estrogen receptors in the experiment was increased, and the effects of vaginal atrophy were reduced, while the activity of osteoblasts was improved [53]. Experimental studies on the effect of placental extracts on behavior and physical condition in the animal model showed decrease in symptoms of fatigue and increased resistance to physical stress [53, 62]. This phenomenon was explained by the rise of the level of intracellular calcium, activation of splenocytes and T cells, and reduction of synthesis of proinflammatory cytokines associated with fatigue (IL6, TNF, and IFN*γ*) [53, 62]. Similar results were obtained in preclinical studies [29].

Placental extracts showed expressed efficiency in neurology by supporting regeneration of the nerve tissue in experimental treatment of the nerve damage and facial spasm. The authors explain the resulting effect with the increased synthesis of GAP-43 and Cdc2 regenerative factors [63, 64]. Placental extracts were effective in the treatment of rheumatoid arthritis [65] and experimental renal failure [66]. A certain amount of practical experience in the application of placental extracts is also accumulated in veterinary medicine. Here, the extracts were applied for stimulation of mammogenesis, lactogenesis, and galactopoiesis [67].

## 6. Cord Blood Serum

Cord blood serum has been used in ophthalmology in treatment of chemical and thermal damage of the cornea and corneal erosion, as well as recovery after laser operations. A more complete and rapid epithelialization of the cornea after application of the preparations containing cord blood serum in comparison to conventional pharmacological therapies was demonstrated [30, 68]. Besides, positive effects in the treatment of neurotrophic keratitis were described [69].

The ability of cord blood serum to stimulate pancreas cells for insulin synthesis along with the formation of pancreatic islets has been demonstrated *in vitro* [70]. Application of cord blood serum in obstetrical antiphospholipid syndrome improves the readiness of preimplantation endometrium and reduces the number of antiphospholipid antibodies [71]. Besides, application of cord blood serum has shown efficiency in wound healing [72, 73]. These effects are explained by the presence of EGF, FGF, HGH, fibronectin, NGF, and IGF-1 in the composition of cord blood serum [68].

## 7. Isolated Placental Cells

By now, cells from the amniotic and chorionic membranes, placental villi, and umbilical cord have been successfully isolated, phenotyped, and characterized [74, 75]. Protocols based on trypsin, collagenase, or dispase digestion are used to isolate the cells from the fetal membranes. Isolation of the cells from placental villi requires application of DNase, and isolation of the cells from umbilical cord requires hyaluronidase or application of the explant technique [6]. Among the variety of cell types, which can be isolated from the placenta and placental derivatives, mesenchymal stromal cells (MSCs) receive the highest attention in research and clinical trials [21]. According to the majority of researchers, MSCs obtained from all placental sources express CD105, CD90, CD73, CD29, CD13, CD10, and to a minor extent HLA-A, B, and C, while not expressing CD14, CD34, CD45, and HLA-DR [21]. Importantly, the cells obtained by the mentioned protocols retain the ability to synthesize chorionic gonadotropin and express cytokeratin-7 and CD200 [14]. Moreover, placental MSCs possess the capability of differentiation not only into three classical mesodermal lineages (adipogenic, osteogenic, and chondrogenic) but were also shown to be able to differentiate in myogenic, angiogenic, pancreatic, cardiogenic, and neurogenic cell types [6, 21].

*In vivo* studies in the mouse model showed that placenta-derived cells inhibit the delayed hypersensitivity reaction, improve the course of experimental encephalomyelitis, and induce tolerogenic immune response due to differentiation of dendritic cells and inhibition of Th1 response in favor of the Th2. Inhibition of the antigen-specific proliferation of T cells was observed *in vitro* [8, 14]. A large number of *in vitro* and *in vivo* studies is devoted to comparative analysis of the influence of mesenchymal stromal cells from various sources on the cells of the immune system. It was shown that the cells derived from the placenta possess more pronounced immunomodulating effect in comparison to the cells of the adipose tissue and bone marrow [76, 77]. Moreover, placental MSCs possess higher proliferation rates in comparison to the cells from the other sources [78, 79], which can be highly valuable in reconstructive therapies (e.g., tissue engineering) [80].

MSCs of amniotic membrane were efficient in the treatment of premature depletion of ovarian function after chemotherapy. Direct administration to the murine ovary resulted in recovery of the estrous cycle and sexual function, as well as certain other reproductive parameters [81].

It was shown that mesenchymal stromal cells isolated from the amniotic membrane can differentiate into hepatocyte-like cells [21, 82]. Intravenous application of placental cells in experimental models of intoxication with carbon trichloride enhanced liver regeneration and accelerated recovery of the animals [83, 84]. Transplantation of placental cells in the heart muscle resulted in their differentiation into cardiomyocytes [85], which improved the regeneration after experimental acute myocardial infarction [31, 86]. Efficiency in the heart failure treatment is attributed to paracrine interactions [87].

Expression of a range of neuronal markers was found in placental cells, possibly due to the involvement of the placenta in the metabolism of neurotransmitters [88]. Neuroprotective effect of placental MSCs has been proven after direct transplantation to the central nervous system as well as after intravenous administration; the course of experimental Parkinson's disease and spinal cord injury has been improved, and acceleration of rehabilitation after experimental ischemic stroke in the animal model was observed [6, 89, 90]. Efficiency of placental cells in the treatment of ischemic stroke is explained by the influence of VEGF, HGF, and neurotrophic factors [91]. Moreover, reduction of beta-amyloid plaques and anti-inflammatory effect with increasing TGF-*β* and IL10 was observed in the treatment of Alzheimer's disease [92].

Treatment of experimental diabetes with placental cells (intravenous administration) resulted in normalization of the weight of the laboratory animals and restoration of normal glucose levels. The authors detected no teratoma formation, at least within the few months of the follow-up observations [93]. Moreover, treatment with placental cells had positive effects on complications of diabetes. Namely, it accelerated wound healing in a diabetic animal model [94].

Antiaging effect of transplantation of placental cells has been reported in a mouse model [95]. Intravenous application of placental cells demonstrated efficiency in the treatment of rare diseases in the experiment, in particular, pulmonary fibrosis, osteogenesis imperfecta, and muscular dystrophy [6, 21, 96].

Besides the cells isolated from the term placental material, immature placental cells from amniocentesis and chorionic villus sampling have been successfully applied in prospective studies [97, 98]. Since the fetus is not sacrificed in these cases, the ethical issues surrounding discarded early gestation placental tissue are avoided.

Cells obtained from prebirth tissues, such as the umbilical cord blood, amniotic fluid, and chorionic villi, have great potential in cardiovascular tissue engineering for the fabrication of heart valves, prevascularization of *in vitro* engineered tissue constructs, or *in vitro* endothelialization of synthetic blood vessel replacements [99, 100]. Combined with the use of cell banking technology, this approach may be also applied for postnatal applications [101].

## 8. Amniotic and Chorionic Membranes

Amniotic membrane has been used in clinical practice to a higher extent in comparison to the other components of the placental complex. Mainly, it is used in surgery as a biological coating [102, 103]. The first reported treatment with the amniotic membrane was performed in 1910 by J. Devis for the closing of skin defects; later, it was applied as the plastic material in different fields of surgery [32]. Most often, the amniotic membrane is used in ophthalmology for the closure of corneal pathology defects. Such application is determined by a range of unique properties of the amniotic membrane, such as transparency and the ability to stimulate proliferation and migration of stem cells from the limbus area, as well as the ability to suppress vascularization [103, 104]. The method is widely applied in clinics with expressed long-term effect [105, 106]. Application of amniotic and chorionic membranes is also convenient for the treatment of nonhealing trophic ulcers, vaginal reconstruction surgery, enterocutaneous fistula, prevention of adhesions, orthopedic pathology, replacement of the pelvic peritoneum, and so on [8, 19, 107, 108]. The mechanism of action of placental membranes is explained by the effect of biological dressings, activation of epithelialization and neovascularization, suppression of inflammation, and scarring, as well as by antimicrobial properties [19, 109].

## 9. Placental Tissues

References on the application of placental fragments are rarely found in the international scientific literature databases. Successful application of the placental tissue in extracorporeal detoxification was reported [110]. The placental tissue has been used as a biosorbent for the treatment of chronic inflammatory diseases (peritonitis, suppurative cholangitis, mastitis, pancreatic necrosis, and phlegmon). The treatment resulted in improved general condition, reduction of bacteria in blood of the patients, lowering of the laboratory indicators of endotoxemia (bilirubin by 56%, transaminases by 55%, and creatine by 25%), positive dynamics in parameters of the immune system, strengthening of the central and peripheral blood flow, and, importantly, the absence of traumatization of cells in comparison to other conventional sorbents [110]. The efficiency of application of cryopreserved placental fragments was shown in experimental atherosclerosis: acceleration of atherosclerosis regression and neoangiogenesis of myocardium were observed [111]. Utilization of placental fragments in male infertility allowed increasing the quality of sperm as well as the number of pregnancies in couples [112].

## 10. Amniotic Fluid

Amniotic fluid is rarely used in experimental and clinical studies compared to the other placental material due to a smaller number of cells and the active compounds, as well as due to the difficulty of obtaining under sterile conditions. Nevertheless, certain researchers reported the efficiency of the amniotic fluid in bone healing [113], regeneration of nerve tissue [114], and prevention of epidural fibrosis [115]. Recently, an interest in amniotic fluid as a source of stem cells has increased due to a potential in the correction of pathologies of the nervous, musculoskeletal, reproductive, and cardiovascular systems [99, 116, 117]. Preclinical studies in the ovine animal model showed the possibility of prenatal implantation of tissue-engineered cell-based heart valves, composed of biodegradable matrixes with autologous amniotic fluid cells. Such valves possessed *in vivo* functionality with intact valvular integrity and absence of thrombosis [118]. Moreover, amniotic fluid cells have proven high efficiency in neurology: successful spina bifida treatment was performed in both rodent and ovine animal models [97, 119].

## 11. Current Research and Clinical Trials

The areas of research and the number of studies and clinical trials utilizing placental derivatives were analysed on the basis of open sources of the US National Library of Medicine (https://www.ncbi.nlm.nih.gov) and the US National Institutes of Health (http://ClinicalTrials.gov/) (Table 1). Currently, the highest number of studies and clinical trials is dedicated to *cord blood cells*. As hematopoietic cells, they are traditionally used in the treatment of blood oncology and bone marrow pathologies. Novel studies focus on rehabilitation after chemotherapy, diabetes, and ischemic lesions of the central nervous system and extremities. *MSCs* of placental origin have higher differentiation potential but require more complicated isolation procedures. The number of experimental studies and clinical trials with placental MSCs is generally smaller in comparison to cord blood cells; however, the range of pathological conditions where they can be effectively applied is broader. Among others, this includes autoimmune and endocrine diseases, disorders of the nervous and reproductive systems. Most of the works on MSCs are fundamental and experimental; the number of clinical trials is relatively small. Research dedicated to *amniotic membranes* mainly relates to the coverage of wounds, surgical defects, ulcers, and burns. Fewer studies are dedicated to the application of the amnion in the cell culture. At the same time, the number of publications on clinical application of the amnion is significantly higher in comparison to the basic research. The number of studies on *placental extracts* is relatively low and is mainly associated with degenerative diseases and disorders of the reproductive system. Works on application of *cord blood serum* are mainly performed in corneal pathology. Despite the widespread usage of fetal serum as a rich source of growth factors for the cell culture techniques, the intensity of cord blood serum-associated research is rather low.

## 12. Biobanking of Placental Components

Active work with biological material requires appropriate biobanks and biobanking technologies. Long-term storage of placental components may be aimed at clinical application and scientific research, as well as development and testing of the novel drugs [3, 120–123].

Methods of chemical preservation, high-temperature sterilization, hypothermic storage, low-temperature storage, and cryosublimation have been used for the storage of placental components. Selection of the storage method depends on the type of material and the purpose of its further application [5, 120, 122, 124].

Methods of sterilization by filtration or autoclaving have been used for devitalization of the extracts containing peptides [4]. Devitalization methods allow storing the material at the room temperature without additional equipment; however, the properties of such biological objects are significantly altered. For example, devitalization of amniotic membrane significantly reduces the immunogenicity and highly extends its biodegradation period [3].

Hypothermic and subnormothermic storage of biomaterial ensures preservation for a short period of time, required for delivery of the material to a laboratory or clinics with minor structural and biochemical injuries [122, 125–127].

The most conventional method for storage of biological objects, which provides high levels of preservation for a long period of time, is cryopreservation [124]. Cord blood serum, placental extracts, cell suspensions, chorionic and amniotic membranes, and placental tissue are all suitable for cryopreservation procedures [120, 123]. Possibility to isolate MSCs with a stable genome from cryopreserved placental tissues, similar to the population of cells isolated from the native (fresh) tissues, significantly extends the scopes of cryopreservation of the placental material [128]. The placenta is a unique object for low-temperature biobanking and autobanking [6, 120, 122]. In most countries, the application of placenta does not face ethical issues and women positively evaluate this possibility, otherwise considering the material as a “waste.” Donation of the placenta is physiologically indifferent for the donor. At the same time, it provides a large amount of material suitable for direct application and for long-term storage in initial state or after processing, as well as for the preparation of extracts and isolation of cells or individual components [3]. Biobanking technologies might offer a lifelong availability of the autologous placental material or tissue-engineered constructs, readily available for immediate application for a patient [101].

## 13. Conclusions

Considerable amount of information on the properties, experimental studies, and possibilities of clinical application of placental components is accumulated to date (Table 1). The researchers showed the potential use of placental components in various fields of biology and medicine. However, many findings are not confirmed independently by different research groups and have not been performed on the amount of material sufficient enough for clinically significant statistical conclusions.

Early works were primarily devoted to the study of placental extracts as hormonal agents and biostimulators, as well as amniotic membranes as biocovers. Cord blood cells are the most widely used placental component in modern medicine, being applied in the stem cell transplantation. Amniotic membrane is successfully applied in the ophthalmic practice, surgery, and wound healing. Novel technologies based on the application of placental MSCs and autobanking are considered as the most promising and prospective for the near future in the field of regenerative and reconstructive therapies as well as in bioengineering. The interest of researches in placental extracts and placental blood serum is still low, despite the widespread application of various fetal sera in the cell and tissue culture media.

Independent research groups at different time points received similar data on the properties and effects of application of the various components of the placenta for correction of a wide spectrum of pathologies. In most of their opinion, general therapeutic properties of various placental components are expressed in stimulation of reparative processes and anti-inflammatory and immunomodulatory effects. The mechanism of action of the various components of the placenta on the recipient's organism is associated with a shift from Th1 to Th2 type of immune response, suppression of the synthesis of IL6, TNF, and IFN*γ*, and enhancement of the synthesis of IL10, VEGF, and trophic factors.

Various research teams have verified the effects from the application of placenta and its derivatives on the nonhealing wounds, ulcers, disorders of the reproductive system (infertility and menopausal syndrome), autoimmune pathologies, and diabetes, as well as neurological disorders. The use of stem cell therapy methods is accepted as an addition rather than an alternative to conventional medical approaches, since it often does not cover all components of the pathogenesis of the targeted diseases.

Considering the clinical potential and high perspectives of the placental material as an object for autobanking, it may be recommended to preserve not only the individual cell populations but also fragments of membranes, tissue, placental extracts, and cord blood serum.

There are no doubts in the conception of placental components as a rich source of biologically active substances and stem cells. At the same time, it should be noted that currently available results on the positive effects of placental components in the treatment of a wide spectrum of diseases have to go through the test of time and statistical observations in order to determine their potential/real therapeutic efficacy. Additionally, adverse effects and contradictions should be carefully and precisely studied and evaluated in the short- and in the long-term periods.

## Figures and Tables

**Table 1:** Worldwide progress in the research, preclinical studies, and clinical application of the placenta-derived material.

Forms of application of placental components	In research (https://www.ncbi.nlm.nih.gov/) 2017	Clinical trials (http://clinicaltrials.gov/) 2017	Pathology
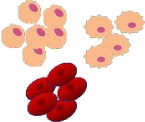 Cord blood cells	>10,000	351	Aplastic anemia, haematological malignancies, cancer, traumatic brain injury, virus infection, limb ischemia, stroke, and brain ischemia

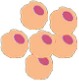 MSCs (derived from placental tissue, fetal membranes, Wharton's jelly, and amniotic fluid)	>4000	39	Diabetes mellitus, multiple sclerosis, myocardial infarction, strokes, peripheral neuropathy, trophic ulcers, Crohn's disease, graft-versus-host disease, and pulmonary fibrosis, limb ischemia, cardiomyopathy, knee osteoarthrosis, diabetes mellitus, amyotrophic lateral sclerosis, and erectile dysfunction

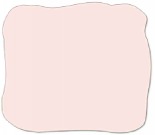 Amniotic membrane	>1900	114	Corneal ulcers, corneal melting, injury, periodontitis, diabetic foot, foot ulcer burns, and adhesions

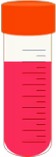 Placental extract	65	2	Keratinocytes, wound healing, rheumatoid arthritis, intervertebral disc degeneration, and climacteric symptoms in premenopausal women

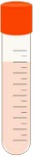 Cord blood serum	7	3	Corneal epithelial wound

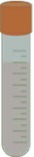 Amniotic fluid	4	—	Preventing fibrosis, adhesion, nerve, and bone regeneration
